# Supplementary material for: Institution and gender-related differences in publication speed before and during COVID-19
Source: PLoS One. 2022 Nov 17;17(11):e0277011. doi: 10.1371/journal.pone.0277011 (PMC9671365; doi:10.1371/journal.pone.0277011)
Supplement: S1 Appendix — (DOCX) [file pone.0277011.s001.docx]

**Appendix**

**Figure S1.** Flowchart of data collection, inclusion, and exclusion criteria for the analysis sample.

The flowchart in Figure S1 summarises the sample construction. The total sample of journals containing COVID-19-related papers (“COVID sample”) was equal to 3122 outlets, with a distribution of papers per journal ranging from a maximum of 737 to a minimum of 1 (Median=4, Mean=11, SD=23.2). To select the minimum number of papers each journal must include we considered different issues related to the bibliometric data generation procedure adopted in this paper. First, since data are clustered at the journal level (because ultimately papers are published in journals), we needed to assure the field comparability of the different outlets, due to differences in publication practices across research fields. Second, we experienced some attrition when matching our PubMed records with article meta-data included in the modified version of WoS. Moreover, this also happened when matching these records with Leiden and QS indicators. Therefore, in line with recent bibliometric literature investigating COVID-19-related sources (1), and expanding on the current thresholds for journal selection found in the literature on scientific publication practices during the pandemic (2–4) we included journals containing a minimum of 50 papers.This resulted in a total sample of 113 journals; these have been classified according to their fields (combining the OECD category scheme for fields of Science and Technology (5), the Web of Science field description and the National Science Foundation discipline specification). Then, we only selected journals publishing in the field of medical, biomedical and health science topics, hence focusing on field-comparable sources. This resulted in a sample of 85 scientific outlets, which was further restricted to 80 journals when matching these data with article meta-data in Web of Science (WoS). During the sample construction process, this meant including only papers published in the 80 journals at the “Data cleaning II” phase (Figure S1). This allowed us to design a comprehensive sample of scientific outlets publishing COVID-19 and not-COVID-19-related research in the medical science field.


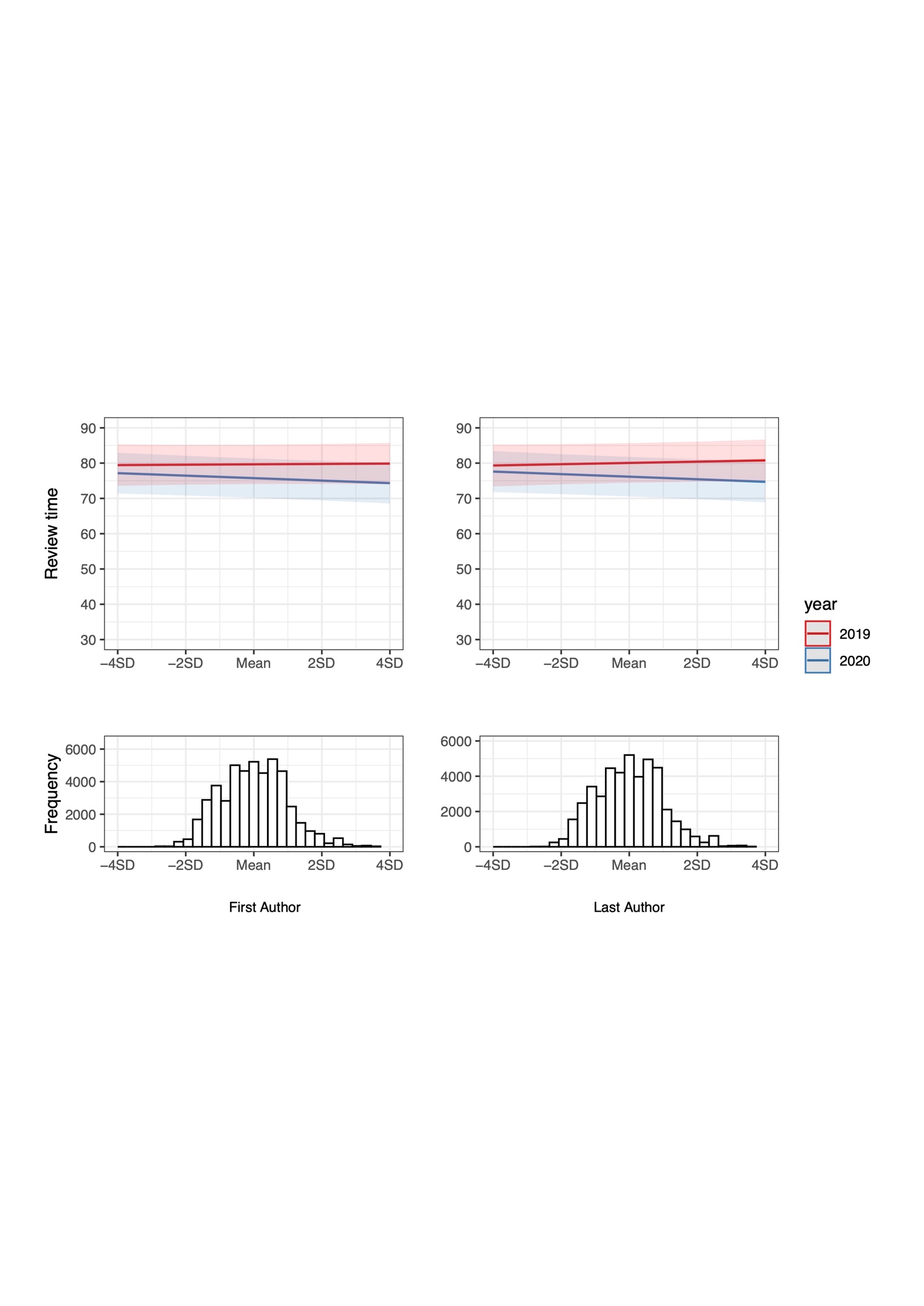


**Figure S2.** Estimated difference in average review time for papers published in 2019 (red line) and 2020 (blue line) by authors from lower and higher ranked universities. The Y-axis reports the number of days from manuscript submission to acceptance. The X-axis scores Institutions status (according to the Leiden Ranking’s PP-top 10% measure) by standard deviations. The light red and light blue areas around the lines indicate the 99% confidence intervals. The histograms below the interaction plots display the frequency distribution of papers across various levels of Institution status


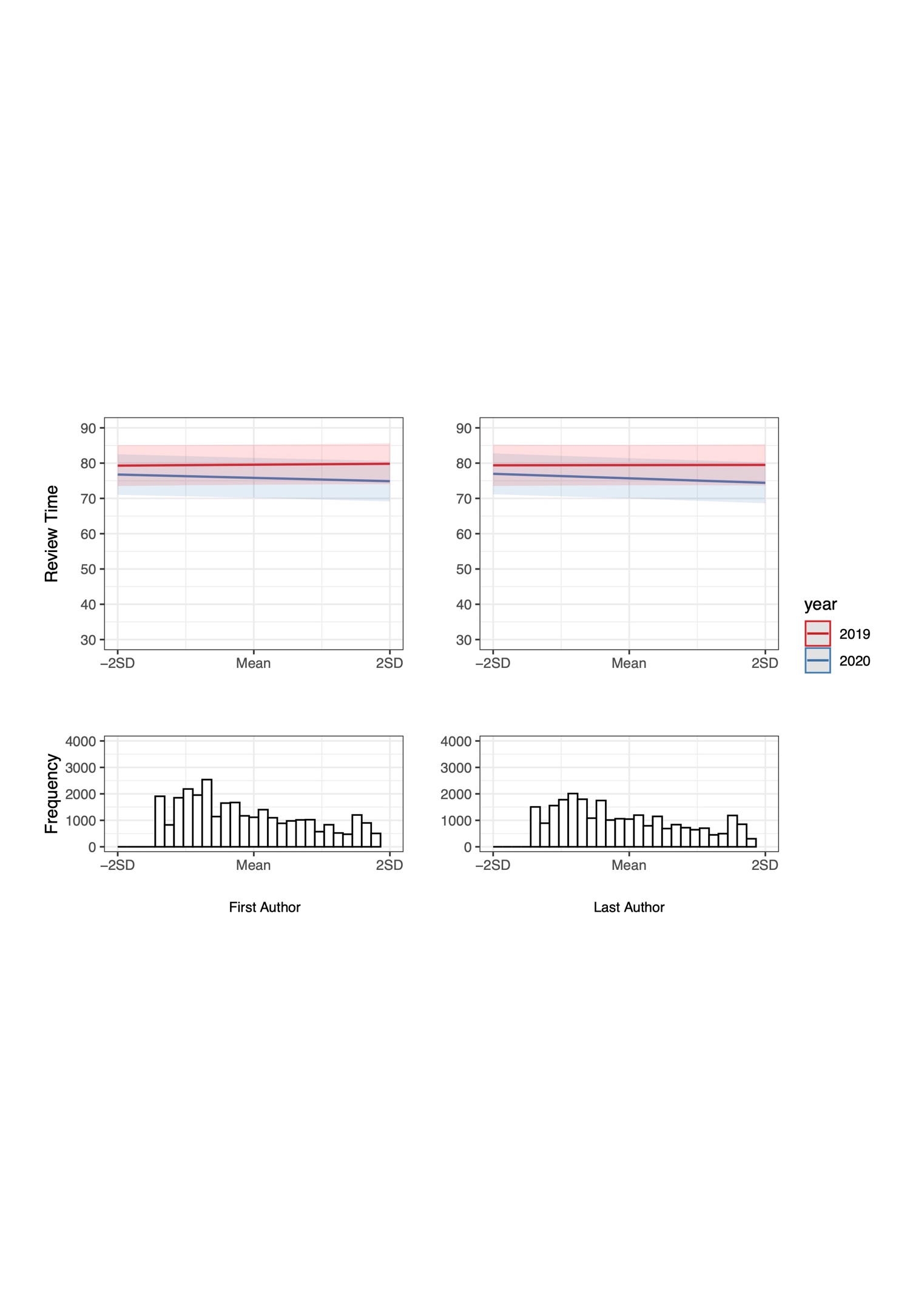


**Figure S3.** Estimated difference in average review time for papers published in 2019 (red line) and 2020 (blue line) by authors from lower and higher ranked universities. The Y-axis reports the number of days from manuscript submission to acceptance. The X-axis scores Institutions status (according to the QS’s full ranking indicators) by standard deviations. The light red and light blue areas around the lines indicate the 99% confidence intervals. The histograms below the interaction plots display the frequency distribution of papers across various levels of Institution status.


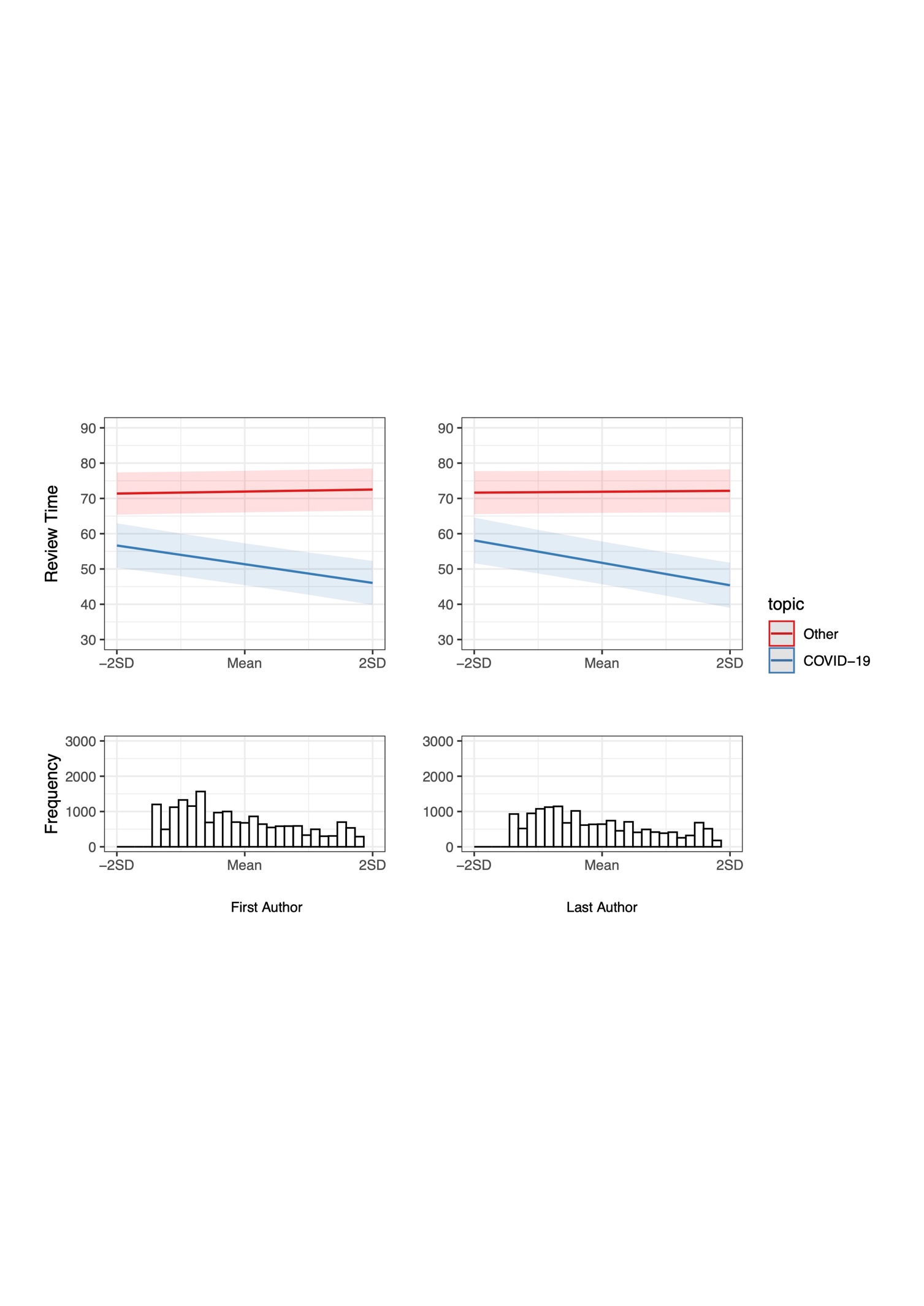


**Figure S4.** Estimated difference in average review time for COVID-19 papers (red line) and research on other topics (blue line) published in 2020 by authors from lower and higher ranked institutions. The Y-axis reports the number of days from manuscript submission to acceptance. The X-axis scores Institution status (according to the QS’s full ranking indicators) by standard deviations. The light red and light blue areas around the lines indicate the 99% confidence intervals. The histograms below the interaction plots display the frequency distribution of papers across various levels of Institution status.

**Figure S5.** Survival plots of time from submission to publication for COVID-19 papers authored at higher-ranked (teal line) *versus* lower ranked universities (red line). The groups of highly ranked and lower ranked universities are inferred from the QS Ranking. Dashed lines in black specify the difference in median review times of papers from highly ranked and lower ranked institutions.

**Figure S6.** Survival plots of time from submission to publication for COVID-19 papers published in single-blind *versus* double-blind peer reviewed journals, authored at higher-ranked (teal line) *versus* lower ranked universities (red line). The groups of highly ranked and lower ranked universities are inferred from the QS Ranking. Dashed lines in black specify the difference in median review times of papers from highly ranked and lower ranked institutions.

**Figure S7.** Survival plots of time from submission to publication for papers on other topics than COVID-9 published in single-blind *versus* double-blind peer reviewed journals in 2020, authored at higher-ranked (teal line) *versus* lower ranked universities (red line). The groups of highly ranked and lower ranked universities are inferred from the Leiden Ranking. Dashed lines in black specify the difference in median review times of papers from highly ranked and lower ranked institutions.

**Figure S8.** Survival plots of time from submission to publication for papers on other topics than COVID-19 published in single-blind *versus* double-blind peer reviewed journals in 2020, authored at higher-ranked (teal line) *versus* lower ranked universities (red line). The groups of highly ranked and lower ranked universities are inferred from the Leiden Ranking. Dashed lines in black specify the difference in median review times of papers from highly ranked and lower ranked institutions.

**Figure S9.** Survival plots of time from submission to publication for papers published in single-blind *versus* double-blind peer reviewed journals in 2019, authored at higher-ranked (teal line) *versus* lower ranked universities (red line). The groups of highly ranked and lower ranked universities are inferred from the Leiden Ranking. Dashed lines in black specify the difference in median review times of papers from highly ranked and lower ranked institutions.

**Figure S10.** Survival plots of time from submission to publication for papers published in single-blind *versus* double-blind peer reviewed journals in 2019, authored at higher-ranked (teal line) *versus* lower ranked universities (red line). The groups of highly ranked and lower ranked universities are inferred from the QS Ranking. Dashed lines in black specify the difference in median review times of papers from highly ranked and lower ranked institutions.

**Figure S11.** Random intercept effect distribution plot. Journal whose average publication speed, measured in days, are marked in blue, while journals with publication speed below the means are marked in red.

**Table S1.** Variable specification

**Table S2.** Mixed linear regression model predicting the change in peer-review duration from 2019 to 2020 (QS Ranking)

| **Linear mixed effect models** | | | | | | | | |
| --- | --- | --- | --- | --- | --- | --- | --- | --- |
|  | **M1 peer-review time**  **(First author)** | | **M2 peer-review time**  **(First author)** | | **M3 peer-review time**  **(Last author)** | | **M4 peer-review time**  **(Last author)** | |
| *Predictors* | *Estimates* | *99%CI* | *Estimates* | *99%CI* | *Estimates* | *99%CI* | *Estimates* | *99%CI* |
| Intercept | 76.27 | 68.66 – 83.87 | 79.67 | 72.18 – 87.16 | 75.76 | 68.06 – 83.47 | 79.21 | 71.66 – 86.77 |
| Institution Status (QS) | 0.93 | -0.55 – 2.40 | 0.27 | -1.18 – 1.72 | 0.68 | -0.90 – 2.26 | 0.06 | -1.49 – 1.61 |
| Year | -6.68 | -8.14 – -5.22 | -3.92 | -5.37 – -2.48 | -5.95 | -7.77 – -4.13 | -3.39 | -5.20 – -1.59 |
| Journal Impact Factor | -0.30 | -7.20 – 6.59 | -0.26 | -7.04 – 6.53 | -1.07 | -8.07 – 5.93 | -0.74 | -7.60 – 6.12 |
| Gender (male) | -0.94 | -2.55 – 0.68 | -1.12 | -2.71 – 0.47 | -0.09 | -1.85 – 1.67 | -0.17 | -1.91 – 1.56 |
| Gender (unknown) | 0.52 | -1.51 – 2.55 | 0.70 | -1.29 – 2.69 | 2.52 | 0.06 – 4.98 | 2.68 | 0.26 – 5.10 |
| Institution Status*Year | -2.05 | -3.92 – -0.17 | -1.22 | -3.06 – 0.62 | -2.05 | -4.06 – -0.04 | -1.33 | -3.31 – 0.64 |
| Year*Gender (male) | -1.11 | -3.18 – 0.96 | 0.21 | -1.82 – 2.25 | -1.21 | -3.47 – 1.05 | -0.45 | -2.67 – 1.78 |
| Year*Gender (unknown) | 0.01 | -2.61 – 2.62 | 0.62 | -1.95 – 3.19 | -1.65 | -4.83 – 1.53 | -0.68 | -3.81 – 2.44 |
| COVID-19 |  |  | -23.00 | -24.74 - 21.26 |  |  | -22.85 | -24.77 – -20.93 |
| **Random Effects** | | | | | | | | |
| σ^2^ | 970.83 | | 935.74 | | 956.56 | | 924.09 | |
| τ_00_ | 646.36 _journal_ | | 626.19 _journal_ | | 653.24 _journal_ | | 626.86 _journal_ | |
| ICC | 0.40 | | 0.40 | | 0.41 | | 0.40 | |
| N | 80 _journals_ | | 80 _journals_ | | 80 _journals_ | | 80 _journals_ | |
| Observations | 30900 | | 30900 | | 26653 | | 26653 | |
| Marginal R^2^ / Conditional R^2^ | 0.008 / 0.405 | | 0.035 / 0.422 | | 0.008 / 0.410 | | 0.033 / 0.424 | |
| AIC | 300600.218 | | 299463.471 | | 258926.281 | | 258005.377 | |

**Table S3.** Mixed linear regression model predicting the moderating effect of the COVID-19 topic on peer-review duration (QS Ranking)

| **Linear mixed effect models** | | | | |
| --- | --- | --- | --- | --- |
|  | **M5 peer-review time**  **(First authors)** | | **M6 peer-review time**  **(Last authors)** | |
| *Predictors* | *Estimates* | *CI* | *Estimates* | *CI* |
| Intercept | 71.66 | 63.93 – 79.38 | 71.71 | 63.86 – 79.56 |
| Institution Status (QS) | 0.56 | -0.71 – 1.82 | 0.26 | -1.08 – 1.61 |
| COVID-19 | -18.28 | -21.04 – -15.52 | -18.58 | -22.07 – -15.09 |
| Gender (male) | -0.29 | -1.62 – 1.05 | -0.03 | -1.47 – 1.41 |
| Gender (unknown) | 1.91 | 0.18 – 3.63 | 1.98 | -0.14 – 4.09 |
| Journal Impact Factor | -0.36 | -7.42 – 6.70 | -1.37 | -8.62 – 5.89 |
| Institution Status* COVID-19 | -5.80 | -8.65 – -2.96 | -6.58 | -9.75 – -3.41 |
| COVID-19 *Gender (male) | -3.47 | -6.88 – -0.05 | -2.82 | -6.82 – 1.19 |
| COVID-19 *Gender (unknown) | -3.49 | -7.87 – 0.89 | 0.51 | -4.79 – 5.80 |
| **Random Effects** | | | | |
| σ^2^ | 842.06 | | 827.53 | |
| τ_00_ | 663.98 _journal_ | | 676.29 _journal_ | |
| ICC | 0.44 | | 0.45 | |
| N | 80 _journals_ | | 80 _journals_ | |
| Observations | 18557 | | 15980 | |
| Marginal R^2^ / Conditional R^2^ | 0.039 / 0.463 | | 0.036 / 0.470 | |
| AIC | 178000.112 | | 153035.337 | |

**Table S4.** Estimated difference in average review time by status LR and QS (C.I. 99%) mixed effect models Poisson distribution

| **Poisson distribution models (LR)** | | | | | | |
| --- | --- | --- | --- | --- | --- | --- |
|  | **M1_D peer-review time**  **(First authors)** | | **M2_D peer-review time**  **(First authors)** | | **M5_D peer-review time**  **(First authors)** | |
| *Predictors* | *Incidence Rate Ratios* | *99%CI* | *Incidence Rate Ratios* | *99%CI* | *Incidence Rate Ratios* | *99%CI* |
| Intercept | 71.58 | 63.34 – 80.90 | 75.06 | 66.63 – 84.56 | 64.44 | 55.30 – 75.09 |
| Institution Status (LR) | 1.00 | 1.00 – 1.01 | 0.99 | 0.99 – 1.00 | 1.00 | 1.00 – 1.01 |
| Year | 0.89 | 0.89 – 0.89 | 0.93 | 0.93 – 0.94 |  |  |
| Journal Impact Factor | 1.00 | 0.90 – 1.11 | 1.00 | 0.91 – 1.11 | 1.00 | 0.88 – 1.14 |
| Gender (male) | 0.98 | 0.98 – 0.99 | 0.98 | 0.98 – 0.99 | 1.00 | 0.99 – 1.00 |
| Gender (unknown) | 1.01 | 1.00 – 1.01 | 1.01 | 1.01 – 1.02 | 1.03 | 1.02 – 1.03 |
| Institution Status (LR)*year | 0.98 | 0.98 – 0.99 | 1.00 | 0.99 – 1.01 |  |  |
| Year*Gender (male) | 0.98 | 0.98 – 0.99 | 1.00 | 1.00 – 1.01 |  |  |
| Year*Gender (unknown) | 1.02 | 1.01 – 1.02 | 1.02 | 1.01 – 1.02 |  |  |
| COVID-19 |  |  | 0.68 | 0.68 – 0.69 | 0.73 | 0.72 – 0.74 |
| Institution Status (LR) * COVID-19 |  |  |  |  | 0.93 | 0.92 – 0.94 |
| COVID-19 *Gender (male) |  |  |  |  | 0.94 | 0.93 – 0.95 |
| COVID-19 *Gender last (unknown) |  |  |  |  | 1.00 | 0.98 – 1.01 |
| **Random Effects** | | | | | | |
| σ^2^ | 0.02 | | 0.02 | | 0.02 | |
| τ_00_ | 0.17 _journal_ | | 0.16 _journal_ | | 0.27 _journal_ | |
| ICC | 0.92 | | 0.92 | | 0.94 | |
| N | 80 _journals_ | | 80 _journals_ | | 80 _journals_ | |
| Observations | 48269 | | 48269 | | 28970 | |
| Marginal R^2^ / Conditional R^2^ | 0.020 / 0.922 | | 0.081 / 0.923 | | 0.056 / 0.943 | |
| AIC | 945346.000 | | 920139.407 | | 534997.282 | |

| **Poisson distribution models (QS)** | | | | | | |
| --- | --- | --- | --- | --- | --- | --- |
|  | **M1_D_ QS peer-review time**  **(First authors)** | | **M2_D_ QS peer-review time**  **(First authors)** | | **M5_D_QS peer-review time**  **(First authors)** | |
| *Predictors* | *Incidence Rate*  *Ratios* | *99%CI* | *Incidence Rate*  *Ratios* | *99%CI* | *Incidence Rate Ratios* | *99%CI* |
| Intercept | 70.89 | 62.27 – 80.71 | 74.29 | 65.50 – 84.26 | 63.42 | 53.96 – 74.55 |
| Institution status (QS) | 1.01 | 1.00 – 1.01 | 1.00 | 0.99 – 1.00 | 1.00 | 1.00 – 1.01 |
| Year | 0.89 | 0.89 – 0.90 | 0.94 | 0.93 – 0.94 |  |  |
| Journal Impact Factor | 1.01 | 0.89 – 1.13 | 1.01 | 0.90 – 1.13 | 1.02 | 0.88 – 1.18 |
| Gender (male) | 0.98 | 0.98 – 0.99 | 0.98 | 0.98 – 0.99 | 1.00 | 0.99 – 1.00 |
| Gender (unknown) | 1.01 | 1.00 – 1.01 | 1.01 | 1.00 – 1.02 | 1.03 | 1.02 – 1.04 |
| Institution Status (QS)*year | 0.98 | 0.97 – 0.99 | 0.99 | 0.98 – 1.00 |  |  |
| Year*Gender (male) | 0.98 | 0.97 – 0.99 | 1.00 | 0.99 – 1.01 |  |  |
| Year*Gender (unknown) | 1.01 | 0.99 – 1.02 | 1.02 | 1.01 – 1.03 |  |  |
| COVID-19 |  |  | 0.68 | 0.67 – 0.68 | 0.73 | 0.72 – 0.74 |
| Institution Status* COVID-19 |  |  |  |  | 0.91 | 0.90 – 0.93 |
| COVID-19 *Gender (male) |  |  |  |  | 0.93 | 0.91 – 0.94 |
| COVID-19 *Gender (unknown) |  |  |  |  | 0.96 | 0.94 – 0.98 |
| **Random Effects** | | | | | | |
| σ^2^ | 0.02 | | 0.02 | | 0.02 | |
| τ_00_ | 0.19 _journal_ | | 0.18 _journal_ | | 0.30 _journal_ | |
| ICC | 0.93 | | 0.92 | | 0.94 | |
| N | 80 _journals_ | | 80 _journals_ | | 80 _journals_ | |
| Observations | 30900 | | 30900 | | 18557 | |
| Marginal R^2^ / Conditional R^2^ | 0.018 / 0.929 | | 0.076 / 0.930 | | 0.055 / 0.948 | |
| AIC | 606412.300 | | 589233.321 | | 340969.955 | |

**Table S5.** Estimated difference in average review time by status LR and QS (C.I. 99%) fixed effects with clustered standard error (at journal level) models

| **Fixed effect clustered S.E. (LR)** | | | | | | |
| --- | --- | --- | --- | --- | --- | --- |
|  | **M1_E peer-review time**  **(First authors)** | | **M2_E peer-review time**  **(First authors)** | | **M5_E peer-review time**  **(First authors)** | |
| *Predictors* | *Estimates* | *99%CI* | *Estimates* | *99%CI* | *Estimates* | *99%CI* |
| Institution Status (LR) | 0.60 | -1.27 – 2.48 | 0.11 | -1.66 – 1.87 | 0.59 | -0.56 – 1.73 |
| Year | -6.77 | -11.07 – -2.48 | -4.09 | -7.00 – -1.17 |  |  |
| Gender (male) | -0.87 | -2.77 – 1.02 | -1.05 | -2.89 – 0.80 | -0.27 | -1.41 – 0.88 |
| Gender (unknown) | 0.71 | -1.61 – 3.03 | 0.96 | -1.31 – 3.23 | 1.81 | -0.14 – 3.77 |
| Institution Status (LR)*year | -1.98 | -4.66 – 0.71 | -0.83 | -3.14 – 1.48 |  |  |
| Year*Gender (male) | -1.01 | -3.26 – 1.23 | 0.23 | -1.81 – 2.27 |  |  |
| Year*Gender (unknown) | 0.45 | -2.28 – 3.18 | 0.46 | -2.05 – 2.97 |  |  |
| COVID-19 |  |  | -22.55 | -33.25 – -11.85 | -18.01 | -28.36 – -7.66 |
| Institution Status (LR) * COVID-19 |  |  |  |  | -5.49 | -11.03 – 0.05 |
| COVID-19 *Gender (male) |  |  |  |  | -3.18 | -6.76 – 0.40 |
| COVID-19 *Gender (unknown) |  |  |  |  | -1.96 | -6.92 – 3.00 |
| Observations | 48269 | | 48269 | | 28970 | |
| R^2^ | 0.441 | | 0.460 | | 0.463 | |

| **Fixed effect clustered S.E. QS** | | | | | | |
| --- | --- | --- | --- | --- | --- | --- |
|  | **M1_E_ QS peer-review time**  **(First authors)** | | **M2_E_ QS peer-review time**  **(First authors)** | | **M5_E_QS peer-review time**  **(First authors)** | |
| *Predictors* | *Estimates* | *99%CI* | *Incidence Rate*  *Ratios* | *99%CI* | *Estimates* | *99%CI* |
| Institution Status (QS) | 0.92 | -1.23 – 3.06 | 0.26 | -1.87 – 2.38 | 0.54 | -1.14 – 2.21 |
| year | -6.67 | -10.91 – -2.44 | -3.92 | -6.84 – -1.01 |  |  |
| Gender (male) | -0.94 | -3.23 – 1.36 | -1.12 | -3.36 – 1.13 | -0.28 | -1.78 – 1.22 |
| Gender (unknown) | 0.51 | -2.21 – 3.23 | 0.70 | -1.92 – 3.32 | 1.89 | -0.81 – 4.60 |
| Institution Status (QS)*year | -2.04 | -4.88 – 0.79 | -1.22 | -3.68 – 1.24 |  |  |
| Year*Gender (male) | -1.10 | -3.84 – 1.63 | 0.22 | -2.23 – 2.66 |  |  |
| Year*Gender (unknown) | 0.00 | -3.31 – 3.31 | 0.61 | -2.52 – 3.74 |  |  |
| COVID-19 |  |  | -22.99 | -33.44 – -12.55 | -18.25 | -28.33 – -8.18 |
| Institution Status (LR) * COVID-19 |  |  |  |  | -5.82 | -10.45 – -1.19 |
| COVID-19 *Gender (male) |  |  |  |  | -3.44 | -7.45 – 0.57 |
| COVID-19 *Gender (unknown) |  |  |  |  | -3.51 | -9.05 – 2.03 |
| Observations | 30900 | | 30900 | | 18557 | |
| R^2^ | 0.449 | | 0.469 | | 0.474 | |

1. Barakat AF, Shokr M, Ibrahim J, Mandrola J, Elgendy IY. Timeline from receipt to online publication of COVID-19 original research articles [Internet]. medRxiv. medRxiv; 2020 [cited 2021 Apr 28]. p. 2020.06.22.20137653. Available from: https://doi.org/10.1101/2020.06.22.20137653

2. Aviv-Reuven S, Rosenfeld A. Publication Patterns’ Changes due to the COVID-19 Pandemic: A longitudinal and short-term scientometric analysis. arXiv [Internet]. 2020 Oct 6 [cited 2021 Apr 19]; Available from: http://arxiv.org/abs/2010.02594

3. Horbach SPJM. No time for that now! Qualitative changes in manuscript peer review during the Covid-19 pandemic. Res Eval [Internet]. 2021 Jan 5 [cited 2021 May 3]; Available from: https://academic.oup.com/rev/advance-article/doi/10.1093/reseval/rvaa037/6064166

4. Homolak J, Kodvanj I, Virag D. Preliminary analysis of COVID-19 academic information patterns: a call for open science in the times of closed borders. Scientometrics [Internet]. 2020 Sep 1 [cited 2021 Apr 29];124(3):2687–701. Available from: https://doi.org/10.1007/s11192-020-03587-2

5. OECD. Frascati Manual 2015 [Internet]. OECD; 2015 [cited 2019 Oct 24]. (The Measurement of Scientific, Technological and Innovation Activities). Available from: https://www.oecd-ilibrary.org/science-and-technology/frascati-manual-2015_9789264239012-en
