# Supplementary material for: Institution and gender-related differences in publication speed before and during COVID-19
Source: PLoS One. 2022 Nov 17;17(11):e0277011. doi: 10.1371/journal.pone.0277011 (PMC9671365; doi:10.1371/journal.pone.0277011)
Supplement: Attachment — Submitted filename: Response to reviewers.pdf [file pone.0277011.s003.pdf]

Dear Reviewers,

Thanks to all of you for your valuable comments and suggestions. These have clearly improved the paper. Reviewer 1-3 found the previous submission ready for publication. Reviewers 3 and 4 had a few smaller requests for edits that we address below.

Comments	Responses
**Reviewer #3:**	
I have no more comments to add, aside from minor fixes on page 4:<br><br>Introduction, sentence "Using recently available data on the peer-review duration of 78,085 medical papers", it's customary not to present N's in this section	Thank you for this suggestion, we have now removed the sample size from the introduction section.
Materials and methods, final sample of COVID-19 papers should be consistent (8848 stated first, then 8828).	Thanks for highlighting these inconsistencies in the materials and methods section. The typo has now been corrected and the sample size of COVID-19 papers is now consistent across the paper.
**Reviewer #4:**	
Although most of the comments are considered, there are some concerns as following.<br>1. Needs to language editing. For example, page 5, line 2: "for further specifications on the underlying methodology, see (43)".	Thank you for this suggestion. We now have updated P4, line 1.
2.In the last paragraph of the introduction, it is repeated aims. Please rewrite this paragraph and summarize.	Thank you for raising this point. We have changed the last paragraph of the introduction by removing the double references to the aim of the study:<br><br>P3, line 33: "In this study, we adopt a new perspective on the widening disparities during COVID-19 by using recently available data on the peer-review duration of metical papers. We aim to examine whether the pandemic has widened

	gender and institution-related differences in the average time from manuscript submission to acceptance. Additionally, given that some peer review processes differ in terms of whether institutional status queues and/or gender are visible to reviewers (due to having either single or double blinded review), we also descriptively investigate whether double blind peer review reduces institution/gender biases in publication speed during the pandemic.”
3.Please clarify, finally, you considered data of "gender unknown", and its results in figures, and interpretation of data or not? If your answer is "yes", it is better to exclude the data, because your main aim is determining the effect of gender on publish speed. If your answer is "NO", that is OK.	We think there may have been some confusion. We are not interested in the analysis of the “gender unknow” category. The paper included this category to avoid excluding from the analysis all the authors for which the three name-to-gender assignment algorithms did not provide reliable results. Indeed, despite for these authors the gender category was “unknown” (hence not discussed as a relevant category in our gender analysis); these authors were still included in the institution-related difference analysis. This allowed to maintain a full sample size in the analysis also for authors whose gender wasn’t captured by the algorithm in the analysis. Therefore, to answer your primary question we are not considering the category “gender unknown” in our analysis. As confirmed by the results and discussion section, no reference to this category is included in the presentation and discussion of the findings. We now include a further clarification about this in the paper P5, line: 18.
4.As there are several papers in the field, what is the novelty of your study? In this regard, I suggested compare your results with the others.	Thank you for this suggestion. The novelty of the paper lies in analysing the widening disparities during COVID-19 by examining changes in the duration of the peer-review process for women and men, and for scientists at different strata of the institutional prestige hierarchy. Additionally, we also descriptively investigate whether double blind peer review reduces institution/gender biases in publication speed during the pandemic. As stated in the paper:<br><br>P11, line 16: “The current study aimed to assess whether the pandemic had widened existing institution and gender-related differences in publishing speed, by using bibliometric metadata on the duration of peer-review. While prior studies have used such data to document increases in the average publication speed during COVID-19 (Else, 2020; Helliwell et al., 2020; Kun, 2020; Zhang et al., 2020), none have examined how changes in journal turn-around times varied by gender and institution status.”.<br><br>In our previous resubmission, we extended the literature review and discussion to cover the relevant papers suggested by reviewers 1, 3 and 4. Moreover, we included additional

	studies published after our initial submission. Since this paper is an empirical study (and not a review study), we cannot cover all existing bibliometric papers on COVID-19. This literature is huge and still growing. Instead, we have limited our focus to published work of direct relevance to our research question. We welcome any further suggestions on literature that directly relates to our research question but feel confident that we have covered all central contributions.
