# Supplementary material for: Institution and gender-related differences in publication speed before and during COVID-19
Source: PLoS One. 2022 Nov 17;17(11):e0277011. doi: 10.1371/journal.pone.0277011 (PMC9671365; doi:10.1371/journal.pone.0277011)
Supplement: Attachment — Submitted filename: Letter to reviewers.docx [file pone.0277011.s002.docx]

We would like to extend our thanks to the reviewers for their encouraging words as well as for the constructive suggestions. To address the reviewers’ concerns, we have made a number of changes to the manuscript. As suggested by different reviewers:

- The aim of the study has been highlighted in the introductory section of the paper
- The references have been restructured and now they are consistently presented in Vancouver stile
- We extended the discussion session and included some of the relevant references suggested by the reviewers
- The appendix has been revised to further clarify sampling procedures

Below, please find point-by-point responses to the reviewer comments. We also made a small number of language changes to the text, marked in the main text in green.

| **Comments** | **Responses** |
| --- | --- |
| **Reviewer #1:** | |
| Overall: The manuscript is about disparities in COVID-19 scientific publication. Although the methodology and statistical analysis was robust and solid, there is a considerable room for improvement in the discussion section.  Introduction: 1. While the general storyline of the Introduction is firm and great, I recommend to transfer the exact aim of the study to last paragraph rather than current first paragraph | Thank you for this suggestion, we have now added the aim of the study to the last paragraph of the introduction:  P3, line 33: “In this study, we adopt a new perspective on the widening disparities during COVID-19 by examining changes in the duration of the peer-review process for women and men, and for scientists at different strata of the institutional prestige hierarchy. Using recently available data on the peer-review duration of 78,085 medical papers, we aim to examine whether the pandemic has widened gender and institution-related differences in the average time from manuscript submission to acceptance. Additionally, given that some peer review processes differ in terms of whether institutional status queues and/or gender are visible to reviewers (due to having either single or double blinded review), we also descriptively investigate whether double blind peer review reduces institution/gender biases in publication speed during the pandemic.” |
| 2. It is suggested to enrich references 11/12 with a comprehensive study on COVID-19 bibliometrics analysis of countries with regard to their income classification: | Thanks for pointing this out. We now have added the relevant references to the text. (Ref. 12). |
| Materials and methods: 1. The numbers on retrieved, excluded, and included papers are kind of a result. Please transfer them to the first paragraph of the results section and make sure there is no overlap between sections. | We appreciate this suggestion; however, we disagree that these values represent results. Instead, they describe the building blocks necessary for the creation of the dataset on which the analysis was then conducted and as such we believe these are more “at home” in the methods section. In spite of their descriptive relevance, these values in of themselves do little to answer the primary research question of the paper. |
| 2. Please kindly provide the reference of mentioned reliability statistics fir name-to-gender assignment algorithms. | We think there may have been some confusion as we do not explicitly state reliability statistics for the name-to-gender assignment. Instead, we write how many of our authors we were able to estimate using an existing database of researcher names to genders. We have attempted to make the text clearer.  P 4, line 32: “The CWTS version of the WoS database also provides an estimated gender for individual researchers based on three name-to-gender assignment algorithms (Gender API, Gender Guesser and Genderize.io). This approach estimates a given author’s likelihood of being a man or a woman based on full names and country affiliations, and has been shown to be ~96% accurate (for further specifications on the underlying methodology, see (Boekhout et al., 2021)). We were able to use this database to assign an estimated gender of 90% of the single authors, 71% of the first authors and 82% of the last authors.” |
| 3. In paragraph on covariates, having COVID-19 keyword or not is more of a binary variable rather than dummy. | Thank you for this suggestion. We now use ‘binary variable’ instead of ‘dummy variable’. |
| Results/Figures/Tables: 1. Please increase the text size in figures, including axis title, legends, and scales. | Thanks for pointing this out, we now have increased the text size in all the Figures and Tables. |
| Discussion: 1. Forth paragraph of the discussion section: The urgent need for rapid research sharing and information dissemination might be generalized to other pandemics as well. It is recommended to broaden your study implications with emphasizing on this generalization and even escalation. For example it is shown in https://doi.org/10.1186/s12889-020-10116-6 that this publication avalanche with probably biased peer-review process is worsening through the time (COVID-19 vs SARS vs Ebola). | Thank you for this suggestion. We have substantially revised the discussion section, which now includes a reference to the publication you mentioned. (Reference 57) |
| **Reviewer #2** | |
| This is an interesting study and well written. The methodology was well written and covered all details needed for reproducibility and replicability  Kindly find below some minor comments: In the Abstract, at the results section, “substantial institution-related disparities in review times in favor of authors from highly-ranked institution”. Kindly add “s” to the “institution”. | Thanks for this suggestion. The abstract has been changed in accordance with your suggestion. |
| Using CADTH for the first time, I think it will be appropriate to write it in full like “(Canadian Agency for Drugs and Technologies in Health (CADTH), 2020)”. | Thanks for pointing out this. The organisation refers to itself as CADTH in publications and this is its preferred name (please see the details here ^[1]^). This is the reason we decided to use such an acronym in the current publication too. Nonetheless, we updated the reference format which now combine the official acronym with the extended organisation name. |
| Be consistent with your citation format. On page 3 and other sections, you used (author, year), e.g. (Cai et al. 2021)- whiles the general format was Vancouver. | Thank you for highlighting these inconsistencies in the citation format. The revised version of the paper now includes all citations in the Vancouver style. |
| The introduction should end by stating the aim/ objectives of the study. This seems missing in your current manuscript. | Thank you for this suggestion, we have now added the aim of the study to the last paragraph:  P3, line 33: “In this study, we adopt a new perspective on the widening disparities during COVID-19 by examining changes in the duration of the peer-review process for women and men, and for scientists at different strata of the institutional prestige hierarchy. Using recently available data on the peer-review duration of 78,085 medical papers, we aim to examine whether the pandemic has widened gender and institution-related differences in the average time from manuscript submission to acceptance. Additionally, given that some peer review processes differ in terms of whether institutional status queues and/or gender are visible to reviewers (due to having either single or double blinded review), we also descriptively investigate whether double blind peer review reduces institution/gender biases in publication speed during the pandemic.” |
| First statement in the methodology that is “On January 11, 2021 ……………….and December 31, 2020” needs to be revised to improve readability. | We have now adjusted this paragraph to improve readability.  P4, line 12: “On January 11, 2021, we searched PubMed Medline for papers including COVID-19 related keywords (‘COVID-19’, ‘COVID’, ‘SARS-CoV-2’, ‘severe acute respiratory syndrome coronavirus 2’, ‘2019-nCoV’, ‘2019 novel coronavirus’, ‘Wuhan coronavirus’) (CADTH, 2020) in their titles or abstracts. These articles needed to be published between March 11, 2020 (the date when the World Health Organization declared COVID-19 a global pandemic) and December 31, 2020. ” |
| Page 5: “Year will be used as an interaction…”, this should be in the past tense. | We have corrected this accordingly.  P 5, line 29: “We interacted *Year* with the Gender and Institution Status in Models 1 to 4….” |
| At result section, under survival plots (3.3) further analyses indicate that …………………COVID-19 “than” have colleagues from …….. Kindly delete “than” ….. | We have changed the text accordingly  P10, line 8: “Further analyses indicate that scientists with prestigious affiliations have benefitted slightly more from fast-track peer reviewing during COVID-19 than colleagues from less reputed institutions. ” |
| Same at the above section, in the second statement, “We use survival plots to” should read as “We used survival”. | We have changed the text accordingly  P10, line 8: “In Figure 2, we used survival plots to visualize within-group variability in the publication speed of COVID-19 articles by authors at highly ranked and lower-ranked institutions in 2020” |
| The authors failed to consider the h-indexes of the first and last authors which could affect the findings of the study. Hence, this should be considered one of the limitations of the study. | This is a very important point. We agree that there may be important differences between groups that can influence the data. We have added the following to the discussion:  P13. line 19: “Similarly, lack of individual-level researcher data constrained what was possible in the current paper, but future research may benefit from running matched studies (e.g., using propensity-score matching) or in the optimal case within-subjects studies. This would help to reduce implicit differences between groups of interest.” |
| The discussion should also highlight on how the quality of papers influence the duration of review. Could this be part of the reasons why articles from institutions of higher repute get published faster? | We completely agree with you. Indeed, the updated draft includes a discussion about how the quality of the paper can influence their duration of peer review at P12, line 7-13.  “At the same time, the observed institution-related disparities may be driven by (average) differences in the quality and immediate relevance of COVID-19 papers coming out of more and less affluent institutions. As mentioned earlier, scientists at less affluent research institutions may have lacked the necessary resources, infrastructure and support to adapt quickly to the changing working conditions imposed by COVID-19. This may have lowered the average quality of their submissions, while also making them slower to respond to resubmission requests, hence delaying the duration of the peer-review process”. |
| **Reviewer #3:** | |
| You should be applauded on your efforts to collate this amount of data, and analyze it using appropriate statistical approaches. I have reviewed your paper and provided some suggestions based on the article sections. Introduction 1. You provided the context of the research nicely here, and your aims might be setter placed at the end of the introduction rather than in the first paragraph | Thank you for this suggestion, we have now added the aim of the study to the last paragraph of the introduction:  P3, line 33: “In this study, we adopt a new perspective on the widening disparities during COVID-19 by examining changes in the duration of the peer-review process for women and men, and for scientists at different strata of the institutional prestige hierarchy. Using recently available data on the peer-review duration of 78,085 medical papers, we aim to examine whether the pandemic has widened gender and institution-related differences in the average time from manuscript submission to acceptance. Additionally, given that some peer review processes differ in terms of whether institutional status queues and/or gender are visible to reviewers (due to having either single or double blinded review), we also descriptively investigate whether double blind peer review reduces institution/gender biases in publication speed during the pandemic.” |
| 2. Re-stating your aims in a clearer way might also help the reader tie in the findings in the results and discussion | We completely agree. Indeed, on top of our change to the text addressed in the comment above, we also now reiterate our aims in the first paragraph of the discussion.  P11, line 16: “The current study aimed to assess whether the pandemic had widened existing institution and gender-related differences in publishing speed, by using bibliometric metadata on the duration of peer-review. While prior studies have used such data to document increases in the average publication speed during COVID-19 (Else, 2020; Helliwell et al., 2020; Kun, 2020; Zhang et al., 2020), none have examined how changes in journal turn-around times varied by gender and institution status.”. |
| Methods The data has been analyzed appropriately by accounting the for clustered nature of the data, i.e. articles clustered within journals. 3. You may wish to indicate what Models 1-5 were in this section | Thank you for raising this point. We now have changed the text in the method section to specify Model 1-6:  P6, line 13: “To measure the relationship between our main predictors (Institution Status and Gender) and the number of days from manuscript submission to acceptance (peer-review duration) we used linear-mixed effects models (West et al., 2014), with papers nested in journals focusing on first author (Model 1; 2; 5) and last author (Model 3; 4; 6) information.”. |
| 4. Have you considered other journal information – e.g. open access, print vs online publication, average publication times? | Thank you for the excellent suggestions. We had not considered these variables explicitly. However, the models controlled for differences between journals by including them as a random effect. Moreover, we also indirectly accounted for such a variation by using journal impact factor as a control variable. We agree these would be interesting variables to extract and analyze directly as moderators of article publication speed. Therefore, we have added this suggestion in the discussion section where we talk about venues for future research.  P 13 line 14: “More generally, there are some areas that research may benefit from expanding upon. For example, while the current study accounted for variations at the journal level through a nested design, we did not directly examine how journal characteristics such as open-access status or print vs. online publication models may have influenced the institutional and gender-related differences observed in our study”. |
| 5. Kaplan-Meier nonparametric estimator plots stratified times to acceptance, unadjusted for other factors. Have you considered using Cox proportional hazards regression to plot adjusted times? | Thank you for this suggestion. We decided not to use the Cox proportional hazards regression model for two reasons. First, in our case the proportional hazard assumptions of the Cox model are violated, so the interpretation of the hazard ratio would have been misleading (Dehbi et al., 2017). Moreover, as we specify in the text (P 6 line 19), as well as in the abstract, we are primarily interested in the descriptive power of survival plots to depict the differences in publication speed between authors affiliated with highly, or lower, ranked universities also across different journal peer review policies. We agree with you that other factors influence peer review time, indeed in our core models we account for journal-level variation in average review times, while adjusting for other factors as well (for more details on that, please refer to results in Table 1 and 2). |
| 6. The benefit of using clustered analysis methods is that you can interpret variation in the outcome by each cluster. Stepwise approach of adding valuables sequentially and looking at the variation in the outcome might be useful to help in explain proportion of variation attributable to journal and author-factors, and then seeing which factor specifically explains the found variation. The paper would benefit from adding interpretation of the random effects in the results section. | Thanks for the excellent suggestions. We see two different points in this comment:  Regarding the issue of interpreting variation of the random effect we have two points 1) We consider differences between journals to be of minor interest when addressing the aim of the paper, which was to assess biases in publishing speed during the pandemic. 2) the sample size per journal becomes fairly small (~100 on average) therefore it becomes impossible to assess meaningful differences between our interactions of interest when comparing across the random effects cluster (i.e. journals). However generally we agree that the article would benefit from some interpretation of the random effects variance, and therefore we had added a new supplementary plot Figure S12 in the appendix, which shows the variation in intercept (i.e. variations in mean publication duration between journals).  Regarding the suggestion of assessing the change in variance explained per fixed effect, we agree this would be beneficial in the case where we wanted to understand the total breakdown of variance between each individual coefficient. However, our modelling setup is instead based on assessing the estimates (and 99% CI) of very specific coefficients of interest (i.e, the interaction between topic, institutional ranking and author’ gender). |
| 7.For gender hypothesis, it would have been interesting to look at the publication rates pre and post COVID by gender, to see if rates for female authors have dropped during COVID times. | Thank you for the interesting suggestion. However, to include this in the current study is not in the scope of the article, as it would require data from all papers published during that time. In addition, there exist numerous studies that show increasing gender gaps in scientific productivity during COVID-19 (Andersen et al., 2020; Cui et al., 2021; Kibbe, 2020; King and Frederickson, 2021; Lerchenmüller et al., 2021; Madsen et al. 2022; Pinho-Gomes et al., 2020; Squazzoni et al., 2021; Vincent-Lamarre et al., 2020). |
| 8.To test whether there is a differential in the duration of peer-review process for women and men the use of propensity matching techniques would have been more appropriate | Thank you for this interesting suggestion. Running a matched study, or even more ideally a within-subjects study, would indeed reduce various biases that may influences our data. We have added this to the discussion as an area for future research in the discussion section at P 13 line 19.  “Similarly, lack of individual-level researcher data constrained what was possible in the current paper, but future research may benefit from running matched studies (e.g., using propensity-score matching) or in the optimal case within-subjects studies. This would help to reduce implicit differences between groups of interest.” |
| 9.Have you considered counting the number of authors, or the corresponding author seniority in the models? (noting that the latter may not be able to be ascertained). Single author papers vs multi-author papers would have different responses to peer reviewer comments. | Thank you for the interesting suggestion. We understand that there could be an indirect effect of being a senior scholar that may be associated with longer experience or a greater number of collaborators. However, this analysis deviates from the primary goal of the paper, that is: “quantifying the differential impact of the pandemic on the duration of the peer-review process for women and men and for scientists at different strata of the institutional-prestige hierarchy”. Moreover, we believe that, since the sample of journals contains both single- and double-blind peer review editorial policies, it would not be possible to grasp the effect that single author papers vs multi-author will have on peer review time. Exactly because for some journals reviewers would know the identity of the authors, while not for other journals. |
| Results Generally well written and supported by tables/figures. 10. Inclusion of a descriptive average/median times by some key factors at the start of the results session can help the reader understand the data better, prior to interpreting model outputs | Thank you for pointing this out. We present a table in the appendix with descriptive statistics for all variables used. However, we noticed that this was not clearly presented in the results. Therefore, we have now added the following to the methods section. P 6 line 9. “Descriptive statistics for all variables can be found in Appendix Table S1.” |
| 11. Inclusions of random effect interpretation would help in describing the variation and explaining which factors drive it. Please see my comment 6. | See our response to comment 6 above. |
| 12. You have excluded a considerable amount of data based on non-assignment of author’s gender. Have you performed analyses on the data without adjusting for gender to see it the institutional disparities exits? | This is a very good observation. However, we have not excluded from our analysis the authors for which the three name-to-gender assignment algorithms did not provide reliable results. Indeed, when you look at Models 1 to 6 you can notice how among the covariates a “Gender unknown” variable is included. This allows to maintain full sample size in the analysis by also including the authors for which the gender disambiguation was not providing satisfying results. |
| Discussion 13. This section could be strengthened by further elaboration of how the results tie in with the existing literature. Gender related differences hypothesis could have benefited from a different analysis, as well as a discussion around what other factors, beyond those measured in the study, could have contributed to the findings | We completely agree with you. The revised paper now includes a deeper discussion on how the results ties with the current literature. P12 line 18  “As for gender, our finding that especially women first-authors (who on average tend to be earlier in their career than women last authors) of COVID-19 papers had a slightly slower publishing speed, align with previous research on gender disparities during the pandemic. According to this literature, lockdowns of schools, universities and daycares took the greatest toll on early career women scientists by considerably reducing their weekly time for research (Breuning et al., 2021; Hengel, 2017; Knobloch-Westerwick et al., 2013; Krukowski et al., 2020; Moss-Racusin et al., 2012; Ross et al., 2006). Further, evidence suggests that initial funding for COVID-19 related research was biased toward male applicants (Breuning et al., 2021) and that women were less likely than men to first-author solicited COVID-19 papers (Brown et al., 2021) . Hence, lower access to resources and fewer (informal) connections to journal editors may also have slowed down early-career women’s publishing speed compared to men’s.” |
| 14. Further discussion about publication rates for covid vs non-covid articles could have been interesting, as well as a discussion about characteristics of COVID published papers. A limitation of the inclusion of 8% (8848/106116) of found COVID papers in the analysis should also be mentioned. | Thank you for pointing this out. Throughout the manuscript, we consistently cite extant evidence on changes in the publications rates and characteristics of COVID-19 research (vs. other research). While these issues are what piqued are initial interest in examining institution and gender-related disparities in publication speed, we consider them to be beyond the scope our study.  We have expanded on the construction of the COVID-19 sample (page 4 section “Materials and methods”) and in Appendix Figure S1. Here we specify that the limitation of the inclusion of 8% of the COVID papers in the sample is due to missing data. Indeed, as reported in Figure S1, the COVID sample size diminishes a first time (e.g., “Data cleaning”), when we select only the sources including submission and acceptance dates. Moreover, the COVID sample is further restricted by the selection of journal article types (e.g., “Data cleaning II”), as explained in the revised Appendix file. While acknowledging the limitations related with data quality, we want to highlight that our sample size of COVID papers expands on the availability of data from comparable studies on academic responses to covid-19 (Helliwell et al., 2020). |
| 15. Discussion should also acknowledge that peer review process is likely to have been impacted by other factors not able to be measured (e.g. editorial load, editorial processes, editor gender, (using the same argument as in the article), number of reviewers reviewing the article etc). | These are excellent points. We agree with you that COVID may also have impacted peer-review policies, practices and processes. We have included these as avenues for future research in the redrafted discussion section. P. 13, line 23å. |
| **Reviewer #4:** | |
| Dear Authors; The manuscript is in interesting field, and well designed. However, it is essential to consider following comments to improve its quality.  -Whole manuscript is required to English language editing. | The manuscript has been edited by a mother language English speaker. Any changes made are highlighted in the main text. |
| -Title should be brief. | Thank you for raising this point. We have now shortened the title. |
| -Please Consider re-building and homogeneous addressing Ref. in the manuscript according to Journal Instruction Authors. | Thank you for highlighting these inconsistencies in the citation format. The revised version of the paper now includes all citation in the Vancouver style. |
| -Please describe the details of all 6 models in method section. | Thank you for raising this point. We have changed the text in the method section to specify Model 1-6:  P6, line 10: “To measure the relationship between our main predictors (Institution Status and Gender) and the number of days from manuscript submission to acceptance (peer-review duration) we used linear-mixed effects models (47), with papers nested in journals focusing on first author (Model 1; 2; 5) and last author (Model 3; 4; 6) information”. |
| Some sections contain un-necessary data such as description on single and blind peer-review. | We apologize about the lack of clarity in the text. Our analysis on how single/double blind peer review interacts with publication biases represents a key part of the paper. To ensure this is clearer, we have added the following to the introduction.  P4. Line 4: “Additionally, given that some peer review processes differ in terms of whether institutional status queues and/or gender are visible to reviewers (due to having either single or double blinded review), we also descriptively investigate whether double blind peer review reduces institution/gender biases in publication speed during the pandemic.” |
| What is your mention of "unknown gender"? If it is really unknown, I suggest to remove their data from the tables. | This is a very good observation. However, we decided to include in our analysis also the authors for which the three name-to-gender assignment algorithms did not provide reliable results. Indeed, when you look at models 1 to 6 you can notice how among the covariates a “Gender unknown” variable is included. This avoids excluding a considerable amount of data based on non-assignment of author’s gender. |
| -As you know, other factors such as "subject area and type of study"; drug discovery, diagnostic tests, treatment approaches, etc have impact on speed of review, and gender disparities (below papers). It is suggested to consider their impact in your models. | This is an interesting suggestion. We have added this as a limitation and avenue for future research.  P13 line 8. “Fourth, differences in study type, not accounted for in our study, may partially explain our findings. For instance, many journal editors likely prioritized speedy reviews of randomized controlled trials during the early-phases of the pandemic. If authors from lower status universities were less likely to take part in such studies, this may have reinforced existing disparities in average review times. Therefore, future studies would likely benefit from breaking down the overall COVID-19 category into specific sub-topics.” |
| -In appendix, it is not necessary description and Ref. | Thank you for the suggestion. We choose to include a description of data collection procedure to supplement Figure S1 in the appendix because we want to enhance the transparency of our sample construction strategy. Indeed, the supporting text further explains some of the relevant choices made during sampling that cannot be included into the figure. Moreover, the Appendix file follows PLOS ONE editorial guidelines. |
| -Discussion should be re-written, needs to more details, compare to previous studies^[2]^, overall needs to deep revision. following studies may be useful, in addition, the current study is not the first study, please correct: | Thank you for this comment. We have expanded on the discussion, referring also to the relevant references you suggested. Regarding your second point, we were unclear about what you were referring to as “first”. We are indeed the first to use publication duration metadata to examine changes in institution and gender-related disparities in review times during COVID-19. To make this clearer we have adjusted the first paragraph of the discussion. |
| -Ref. 30 is incomplete. | Thank you for this suggestion, we have now updated this reference |
| -Most of the Ref. are addressed in introduction. Please reduce the numbers. | Thank you for raising this point. We were not aware of any editorial policy regarding the limit on the number of references permitted in the paper. Hence, we think that the introduction of the paper, where the current literature on the topic is presented and discussed represents the appropriate location for these references. However, after the review and extension of the discussion section, further references have been also included in that section. |

Andersen, J.P., Nielsen, M.W., Simone, N.L., Lewiss, R.E., Jagsi, R., 2020. COVID-19 medical papers have fewer women first authors than expected. Elife 9, 1–7. https://doi.org/10.7554/eLife.58807

Boekhout, H., van der Weijden, I., Waltman, L., 2021. Gender differences in scientific careers: A large-scale bibliometric analysis. 18th Int. Conf. Sci. Inf. ISSI 2021 145–156.

Breuning, M., Fattore, C., Ramos, J., Scalera, J., 2021. The Great Equalizer? Gender, Parenting, and Scholarly Productivity During the Global Pandemic. PS Polit. Sci. Polit. 54, 427–431. https://doi.org/10.1017/S1049096520002036

Brown, C., Novick, T.K., Jacobs, E.A., 2021. Gender Disparities in Authorship of Invited Manuscripts During the COVID-19 Pandemic. Women’s Heal. Reports 2, 149–153. https://doi.org/10.1089/whr.2021.0023

CADTH, 2020. Canadian Agency for Drugs and Technologies in Health [WWW Document]. URL https://covid.cadth.ca/literature-searching-tools/cadth-covid-19-search-strings/#covid-19-medline. (accessed 9.29.21).

Cui, R., Ding, H., Zhu, F., 2021. Gender Inequality in Research Productivity During the COVID-19 Pandemic. Manuf. Serv. Oper. Manag. https://doi.org/10.1287/msom.2021.0991

Dehbi, H.M., Royston, P., Hackshaw, A., 2017. Life expectancy difference and life expectancy ratio: Two measures of treatment effects in randomised trials with non-proportional hazards. BMJ 357. https://doi.org/10.1136/bmj.j2250

Else, H., 2020. How a torrent of COVID science changed research publishing - in seven charts. Nature. https://doi.org/10.1038/d41586-020-03564-y

Helliwell, J.A., Bolton, W.S., Burke, J.R., Tiernan, J.P., Jayne, D.G., Chapman, S.J., 2020. Global academic response to COVID-19: Cross-sectional study. Learn. Publ. 33, 385–393. https://doi.org/https://doi.org/10.1002/leap.1317

Hengel, E., 2017. Publishing while Female. Are women held to higher standards? Evidence from peer review., Cambridge working papers in economics. https://doi.org/https://doi.org/10.17863/CAM.17548

Kibbe, M.R., 2020. Consequences of the COVID-19 Pandemic on Manuscript Submissions by Women. JAMA Surg. 155, 803–804. https://doi.org/10.1001/jamasurg.2020.3917

King, M.M., Frederickson, M.E., 2021. The Pandemic Penalty: The Gendered Effects of COVID-19 on Scientific Productivity. Socius 7, 23780231211006976. https://doi.org/10.1177/23780231211006977

Knobloch-Westerwick, S., Glynn, C.J., Huge, M., 2013. The Matilda Effect in Science Communication: An Experiment on Gender Bias in Publication Quality Perceptions and Collaboration Interest. Sci. Commun. 35, 603–625. https://doi.org/10.1177/1075547012472684

Krukowski, R.A., Jagsi, R., Cardel, M.I., 2020. Academic Productivity Differences by Gender and Child Age in Science, Technology, Engineering, Mathematics, and Medicine Faculty During the COVID-19 Pandemic. J. Women’s Heal. 30, 341–347. https://doi.org/10.1089/jwh.2020.8710

Kun, Á., 2020. Time to Acceptance of 3 Days for Papers About COVID-19. Publications 8, 30. https://doi.org/10.3390/publications8020030

Lerchenmüller, C., Schmallenbach, L., Jena, A., Lerchenmueller, M., 2021. Longitudinal analyses of gender differences in first authorship publications related to COVID-19. BMJ Open 11. https://doi.org/10.1136/BMJOPEN-2020-045176

Moss-Racusin, C.A., Dovidio, J.F., Brescoll, V.L., Graham, M.J., Handelsman, J., 2012. Science faculty’s subtle gender biases favor male students. Proc. Natl. Acad. Sci. U. S. A. 109, 16474–9. https://doi.org/10.1073/pnas.1211286109

Pinho-Gomes, A.-C., Peters, S., Thompson, K., Hockham, C., Ripullone, K., Woodward, M., Carcel, C., 2020. Where are the women? Gender inequalities in COVID-19 research authorship. BMJ Glob. Heal. 5, e002922. https://doi.org/10.1136/bmjgh-2020-002922

Ross, J.S., Gross, C.P., Desai, M.M., Hong, Y., Grant, A.O., Daniels, S.R., Hachinski, V.C., Gibbons, R.J., Gardner, T.J., Krumholz, H.M., 2006. Effect of Blinded Peer Review on Abstract Acceptance. JAMA 295, 1675–1680. https://doi.org/10.1001/jama.295.14.1675

Squazzoni, F., Bravo, G., Grimaldo, F., García-Costa, D., Farjam, M., Mehmani, B., 2021. Gender gap in journal submissions and peer review during the first wave of the COVID-19 pandemic. A study on 2329 Elsevier journals. PLoS One 16, e0257919. https://doi.org/10.1371/JOURNAL.PONE.0257919

Vincent-Lamarre, P., Sugimoto, C.R., Larivière, V., 2020. Monitoring Women’s Scholarly Production during the COVID‐19 Pandemic [WWW Document]. Nat. Index.

West, B.T., Welch, K.B., Galecki, A.T., 2014. Linear Mixed Models, Linear Mixed Models. Chapman and Hall/CRC. https://doi.org/10.1201/b17198

Zhang, L., Zhao, W., Sun, B., Huang, Y., Glänzel, W., 2020. How scientific research reacts to international public health emergencies: a global analysis of response patterns. Scientometrics 124, 747–773. https://doi.org/10.1007/s11192-020-03531-4

1. <https://www.cadth.ca/sites/default/files/pdf/CADTH_style_guide_6th_edition.pdf>
2. 1-Scientometric analysis of medical publications during COVID-19 pandemic: the twenty-twenty research boom.
   Aggarwal A, Agosti E, Singh PM, Varshini A, Garg K, Chaurasia B, Zanin L, Fontanella MM. Minerva Med. 2021 Oct;112(5):631-640. doi: 10.23736/S0026-4806.21.07489-9.
   2-Gender Disparities in Authorship of Invited Manuscripts During the COVID-19 Pandemic.
   Brown C, Novick TK, Jacobs EA. Womens Health Rep (New Rochelle). 2021 May 25;2(1):149-153. doi: 10.1089/whr.2021.0023
   3- Characteristics of academic publications, preprints, and registered clinical trials on the COVID-19 pandemic.
   Gianola S, Jesus TS, Bargeri S, Castellini G. PLoS One. 2020 Oct 6;15(10):e0240123. doi: 10.1371/journal.pone.0240123.
   4-Global academic response to COVID-19: Cross-sectional study.
   Helliwell JA, Bolton WS, Burke JR, Tiernan JP, Jayne DG, Chapman SJ. Learn Publ. 2020 Jul 1:10.1002/leap.1317. doi: 10.1002/leap.1317.
   5-Impact of the Early COVID-19 Pandemic on Gender Participation in Academic Publishing in Radiation Oncology.
   Anabaraonye N, Tsai CJ, Saeed H, Chino F, Ekpo E, Ahuja S, Garcia O, Miller RC. Adv Radiat Oncol. 2021 Nov 18;7(2):100845. doi: 10.1016/j.adro.2021.100845.
